# The Potent Oxidant Anticancer Activity of Organoiridium Catalysts**

**DOI:** 10.1002/anie.201311161

**Published:** 2014-03-11

**Authors:** Zhe Liu, Isolda Romero-Canelón, Bushra Qamar, Jessica M Hearn, Abraha Habtemariam, Nicolas P E Barry, Ana M Pizarro, Guy J Clarkson, Peter J Sadler

**Affiliations:** Department of Chemistry, University of WarwickCoventry, CV4 7AL (UK); Warwick Systems Biology Centre, University of Warwick(UK)

**Keywords:** anticancer drugs, biocatalysts, hydride transfer, iridium, reactive oxygen species

## Abstract

Platinum complexes are the most widely used anticancer drugs; however, new generations of agents are needed. The organoiridium(III) complex [(η^5^-Cp^xbiph^)Ir(phpy)(Cl)] (**1-Cl**), which contains π-bonded biphenyltetramethylcyclopentadienyl (Cp^xbiph^) and C∧N-chelated phenylpyridine (phpy) ligands, undergoes rapid hydrolysis of the chlorido ligand. In contrast, the pyridine complex [(η^5^-Cp^xbiph^)Ir(phpy)(py)]^+^ (**1-py**) aquates slowly, and is more potent (in nanomolar amounts) than both **1-Cl** and cisplatin towards a wide range of cancer cells. The pyridine ligand protects **1-py** from rapid reaction with intracellular glutathione. The high potency of **1-py** correlates with its ability to increase substantially the level of reactive oxygen species (ROS) in cancer cells. The unprecedented ability of these iridium complexes to generate H_2_O_2_ by catalytic hydride transfer from the coenzyme NADH to oxygen is demonstrated. Such organoiridium complexes are promising as a new generation of anticancer drugs for effective oxidant therapy.

Three platinum-based anticancer drugs, cisplatin (CDDP), carboplatin, and oxaliplatin (OXA), are involved in nearly 50 % of all anticancer therapies worldwide; however, problems of platinum resistance and undesirable side effects are limiting their future use.^1^ This highlights the need to develop anticancer agents with new mechanisms of action (MoAs). In contrast to the DNA-targeting platinum drugs, some organometallic complexes that seem to be promising^2^ offer the possibility of alternative redox MoAs. Oxidative stress caused by the generation of reactive oxygen species (ROS) is an effective method of killing cancer cells.^3^ ROS are produced in a wide range of physiological processes, in particular by mitochondria, and play valuable roles in cellular signaling. Uncontrolled and excessive production of ROS, or a diminished ability of cells to scavenge ROS, gives rise to oxidative stress and subsequent damage to various cellular components. As cancer cells are often under increased oxidative stress compared to normal cells, which is partially due to abnormal mitochondrial functions, an element of selectivity is achieved when an anticancer agent further increases the level of oxidative stress. Such stress would have a smaller effect on redox control in normal cells.

Organometallic iridium(III) complexes are particularly promising. Complexes of this third-row low-spin transition-metal ion with a 5 d^6^ electron configuration are often thought to be inert. Indeed, [Ir(H_2_O)_6_]^3+^ exchanges ligands on a time scale of hundreds of years.^4^ However, the introduction of a cyclopentadienyl ligand can increase the ligand exchange rate by 14 orders of magnitude.^5^ There is current interest in the design of both inert and labile Ir^III^ complexes as anticancer agents.^6^ The activity of half-sandwich cyclopentadienyl anticancer complexes [(η^5^-Cp^x^)Ir^III^(X∧Y)Cl]^0/+^, where Cp^x^ can be a pentamethylcyclopentadienyl (Cp*), phenyltetramethylcyclopentadienyl (Cp^xph^), or biphenyltetramethylcyclopentadienyl (Cp^xbiph^) moiety, and X∧Y is a chelating ligand, is highly dependent both on the Cp* substituents and on the X∧Y ligand.6a–c These chlorido complexes all hydrolyze rapidly (within minutes at 310 K), including [(η^5^-Cp^xbiph^)Ir(phpy)Cl] (**1**-**Cl**; phpy=2-phenylpyridine), which is one of the most potent complexes.6c

Herein, we show that the monodentate ligand can have a major influence on both chemical reactivity and anticancer potency. We compare the aquation of the chlorido complex **1**-**Cl** with that of the pyridine (py) complex [(η^5^-Cp^xbiph^)Ir(phpy)py]^+^ (**1**-**py**). We investigated their activity towards a wide range of cancer cells and their selectivity for cancer cells over normal cells and used COMPARE analysis to explore the potential MoAs. We related cellular accumulation of iridium and production of ROS in cells to the redox chemistry of the complexes. In particular, we asked whether the ability of the cyclopentadienyl Ir^III^ complexes to accept a hydride from the coenzyme NADH can be linked to ROS production. We demonstrate that organometallic iridium complexes can be used as highly effective, even catalytic, oxidants for the treatment of cancer.

The novel compound **1**-**py**⋅PF_6_ was synthesized from the chlorido analogue **1**-**Cl**, isolated as the PF_6_^−^ salt (Figure 1 a), and fully characterized by ^1^H and ^13^C NMR spectroscopy, ESI-MS, CHN elemental analysis, HPLC (Figure S1), and X-ray crystallography (Figure 1 b; for details see the Supporting Information, Tables S1 and S2).

**Figure 1:**
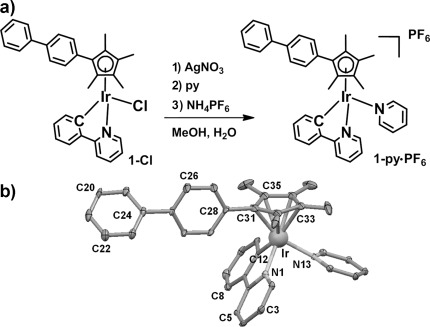
a) Synthesis route for 1-py⋅PF_6_. b) X-ray crystal structure of [(η^5^-Cp^xbiph^)Ir(phpy)py]PF_6_⋅(CH_3_OH)_0.5_,; thermal ellipsoids set at 20 % probability. The H atoms, counterions, and solvent have been omitted for clarity.

First, we assessed the anticancer activity of **1**-**py** in comparison with that of **1**-**Cl** (and cisplatin) and probed their MoAs. We then studied chemical reactions of **1**-**py** that might play a key role in determining its biological activity, especially novel pathways for the production of ROS.

Complex **1**-**py** showed high potency with an IC_50_ value (the concentration at which 50 % of cell growth is inhibited) of 120 nm towards A2780 human ovarian cancer cells, which renders it six times more active than **1**-**Cl**,6c and approximately ten times more active than cisplatin (Figure 2 a and Table S3). Moreover, **1**-**py** is thirteen times less toxic towards normal cells (MRC-5 human lung fibroblast cells) than towards A2780 cancer cells, whereas **1**-**Cl** has a much lower selectivity factor of four (Figure 2 a). Interestingly, the antiproliferative activity of **1**-**py** towards A2780 cells after exposure for four hours is the same as that after 24 hours, which implies that the onset of cell death is a relatively rapid process (Figure S2 and Table S4).

**Figure 2:**
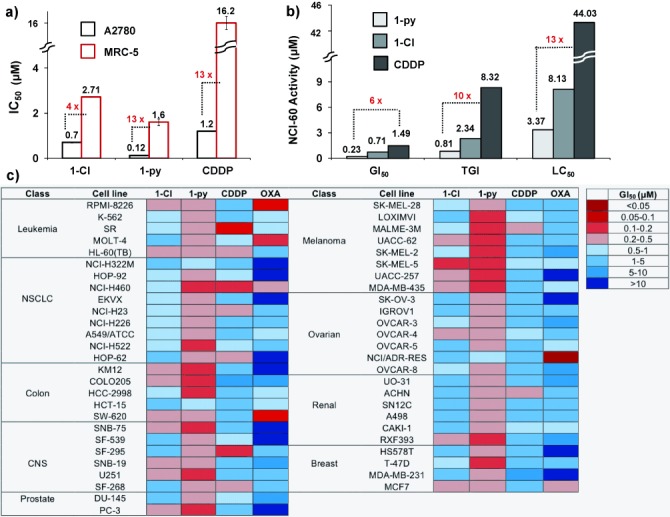
Antiproliferative activity. a) IC_50_ in A2780 cancer cells and MRC-5 normal lung fibroblasts of 1-py, 1-Cl,6c and CDDP. b) GI_50_, TGI, and LC_50_ values (μm) of 1-Cl,^7^ 1-py, and CDDP in the NCI-60 screen. c) Heat map for GI_50_ values of 1-Cl, 1-py, CDDP, and oxaliplatin (OXA). The deep red color corresponds to the highest activity, whereas the deep blue color represents the lowest activity.

The antiproliferative activities of **1**-**Cl**^7^ and **1**-**py** were further evaluated by the National Cancer Institute NCI-60 human cancer cell screen,^8^ which consists of nine tumor subtypes and approximately 60 cell lines (Figure 2 c and Figure S3). Three endpoints were determined: the GI_50_ (the concentration that causes 50 % cell growth inhibition), TGI (concentration that causes 100 % cell growth inhibition), and LC_50_ values (the concentration that decreases the original cell count by 50 %). Complex **1**-**py** is six (GI_50_) to thirteen (LC_50_) times more potent than CDDP and approximately three times more potent than **1**-**Cl** (Figure 2 b). Complex **1**-**py** shows high potency towards a wide range of cancer cell lines (Figure S3), with particular selectivity towards colon, melanoma, and non-small-cell lung cancer (NSCLC). Complex **1**-**py** displayed its highest potency towards the MDA-MB-468 breast cell line with a GI_50_ value of 132 nm. The high potency of **1**-**py** contrasts with the loss of activity when a chloride in a Ru^II^ arene anticancer complex [(η^6^-hexamethylbenzene)Ru(en)Cl]^+^ is substituted by pyridine.^9^

The heat map highlights the distinct differences between the iridium compounds and the platinum drugs (Figure 2 c). Strikingly, **1**-**py** is more active in almost all of the cell lines, and the pattern of selectivity is very different for the iridium and platinum complexes, suggesting different MoAs. We used the NCI COMPARE algorithm, which quantitatively compares the selectivity in the NCI-60 screen of a seed compound with a database of compounds, to produce a Pearson’s correlation coefficient between −1 (negative correlation) and +1 (positive correlation), as a measure of similarity.^7^, ^8^ COMPARE analysis of **1**-**py** showed no correlation to any platinum compounds when we assessed the top 100 correlations with the DTP/NIH synthetics compound database, which hosts more than 40 000 pure, natural and synthetic compounds. This result quantitatively suggests that the MoA of **1**-**py** is different from that of cisplatin and other platinum compounds. In contrast, COMPARE analysis gave a correlation coefficient of 0.744 for **1**-**py** and **1**-**Cl** across the NCI-60 panel, suggesting that they have similar MoAs.

We also investigated the accumulation of complexes **1**-**Cl** and **1**-**py** in A2780 cells. After 24 hours of drug exposure, the amount of iridium that had accumulated in the cells was 20 times larger when complex **1**-**py** was used instead of **1**-**Cl** (8.3±0.3 ng Ir and 0.39±0.05 ng Ir per 10^6^ cells, respectively; Table S3). Uptake of **1**-**py** by A2780 cells was concentration- and time-dependent, rapid in the first 30 minutes, and slowly increasing during the four-hour study (Figure S4).

Some anticancer metal complexes disturb cellular redox homeostasis by increasing the level of oxidative stress.^10^ To assess whether redox chemistry is involved in the MoA, we co-administered **1**-**py** and l-buthionine sulfoximine (l-BSO) to A2780 cells. The tripeptide glutathione (GSH, γ-l-Glu-l-Cys-Gly) is an important antioxidant in cells and a scavenger of ROS. l-BSO, an inhibitor of γ-glutamylcysteine synthetase, is often used to deplete the level of cellular GSH. A two-fold decrease in the IC_50_ value (60±3 nm) was observed upon co-incubation of **1**-**py** with a non-toxic dose of l-BSO (5 μm; Figure S5 and Table S3). These data are consistent with a MoA for **1**-**py** that involves redox processes, as cells are exposed to higher levels of ROS on co-incubation with l-BSO.

To detect changes in general oxidative stress, we determined the levels of ROS in A2780 cells that are induced by **1**-**py** at concentrations of one third of the IC_50_ value, the IC_50_ value, and three times the IC_50_ value by flow cytometry (Figure 3). This allowed the determination of the total level of oxidative stress (combined levels of H_2_O_2_, peroxy and hydroxyl radicals, peroxynitrite, and NO in the FL1 channel), whilst also monitoring superoxide production (in the FL2 channel). All flow-cytometry experiments were conducted with a drug exposure of just one hour, during which **1**-**py** achieved 78 % of its maximum antiproliferative activity (Table S4). We observed a substantial increase in the total ROS (×1230) and superoxide (×700) levels in cells treated with **1**-**py** compared to untreated cells (Figure S6). No significant changes in the ROS level were observed with increased concentrations of **1**-**py**, which suggests that low doses of **1**-**py** (1/3 of the IC_50_) are sufficient to maximize ROS generation. Similar experiments were carried out using **1**-**Cl** and revealed that although **1**-**Cl** also generated ROS, the level of superoxide induction is significantly lower than for **1**-**py** (Figure 3 B). Therefore, the level of ROS induced by the complexes **1**-**py** and **1**-**Cl** correlates with their anticancer activity.

**Figure 3:**
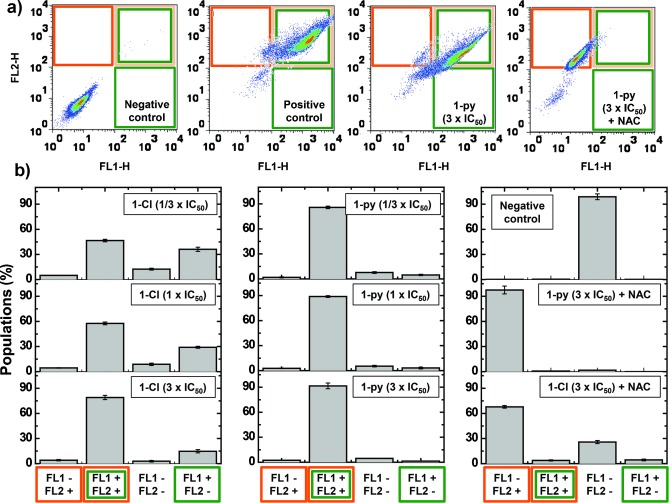
Induction of ROS in A2780 human ovarian cancer cells. a) Comparison of flow cytometry dot plots: Untreated cells (negative control), cells treated with ROS-inducer pyocyanin (1 μm, positive control), cells treated with three times the amount of the IC_50_ value of 1-py, and cells treated with three times the amount of the IC_50_ value of 1-py and NAC (5 μm). The green channel (FL1) detects total oxidative stress, and the orange channel (FL2) detects superoxide production. b) Comparison of the populations exposed to different concentrations (1/3 IC_50_, IC_50_, and 3× IC_50_) of 1-Cl or 1-py and populations exposed to the highest concentration of each iridium complex and NAC. In all cases, the cells were exposed to the drug for one hour at 310 K.

This appears to be the first report of an organometallic iridium anticancer complex that is able to generate significant ROS levels in cancer cells. The highly amplified ROS levels that are induced by **1**-**py** and **1**-**Cl** are likely to play an important role in their activity. Non-enzymatic production of superoxides by xenobiotics has previously been related to the MoAs of organic anticancer drugs such as doxorubicin.^11^

Interestingly, we found that even in the presence of a thiol, such as the ROS scavenger *N*-acetyl-l-cysteine (NAC), the iridium complex is able to cause an increase in the level of superoxide in A2780 cancer cells (Figure 3 b; see also the Supporting Information).

Cancer cells display a redox metabolism that is distinctly different from that of healthy cells.3a Normal cells are able to control ROS levels by balancing the generation and elimination of ROS with appropriate scavenging systems.^12^ To investigate the selectivity of **1**-**py** in terms of the production of ROS in cancer cells versus normal cells, we determined the ROS level in MRC-5 fibroblasts exposed to three different concentrations of **1**-**py**. For cells exposed to an amount of three times the IC_50_ value of **1**-**py**, the population that showed high total oxidative stress as well as high superoxide levels reached only 3.6 % for MRC-5 normal cells (Figure S7), compared to 92.5 % for A2780 cancer cells. Similar observations were made for doses that correspond to a third of the IC_50_ value and the IC_50_ value itself. These results can explain the selectivity of **1**-**py** (the higher potency of **1**-**py** towards A2780 cells compared to MRC-5 fibroblasts; Figure 2 a). Therefore, interference with cellular redox homeostasis in cancer cells appears to play a major role in the MoA of **1**-**py** and provides an attractive approach for cancer therapy.^3^

Next, we investigated the aqueous chemistry of **1**-**py** and, in particular, possible reactions that could produce ROS. First, we studied the hydrolysis (aquation) of **1**-**py**, as this may provide a potential MoA and interaction with possible biological targets. The ^1^H NMR data reveal that the hydrolysis equilibrium was established after four hours at 310 K (63.3 % hydrolyzed, *t*_1/2_=77.8 min; Figure S8). Aquation was reversed when pyridine was added (Figure S8 c). We previously reported that **1**-**Cl** undergoes rapid hydrolysis; this process reached equilibrium within minutes even at 278 K.6c Thus, the introduction of pyridine significantly slows down the hydrolysis rate, which leads to an activation time that is more compatible with transport to biological target sites.

Given the high chloride concentration in the body, we investigated the stability of **1**-**py** in the presence of NaCl (104, 23, and 4 mm), mimicking the Cl^−^ concentration in blood plasma, cell cytoplasm, and cell nucleus, respectively.^13^ After one hour, 7–18 % of **1**-**py** had reacted with chloride to give **1**-**Cl** (Figure S9).

Coenzyme NADH plays a key role in numerous biocatalyzed processes. Previously, we have shown that NADH can donate a hydride to aqua Ir^III^ cyclopentadienyl complexes and induce the reduction of protons to H_2_ and that of quinones to semiquinones.^14^ Now, we have investigated whether reactions of **1**-**py** and **1**-**Cl** with NADH can produce ROS and thus provide a pathway to an oxidant MoA.

When NADH (3.5 mol equiv) was added to a 0.25 mm solution of **1**-**Cl**, a sharp singlet at −14.7 ppm was observed in the ^1^H NMR spectrum within ten minutes; this resonance corresponds to the Ir^III^ hydrido complex [(η^5^-Cp^xbiph^)Ir(phpy)(H)] (**1**-**H**; Figure 4 a). The large upfield shift of this peak compared to that for [(η^5^-Cp*)Ir(phen)(H)]^+^ (ca. −11.1 ppm)14a is notable. NADH was converted into its oxidized form NAD^+^ (new peaks at 8.98, 9.35, and 9.58 ppm assignable to the hydrogen atoms at the C4, C6, and C2 positions of the nicotinamide ring of NAD^+^). These data suggest that **1**-**Cl** can accept a hydride from NADH. Similar results were obtained for the reaction of NADH with **1**-**py** (Figure S10), but the reaction was much slower (a few hours), perhaps because of the difference in hydrolysis rates of the two iridium complexes. Strikingly, data from UV/Vis spectroscopy suggested that **1**-**Cl** and **1**-**py** can act as catalysts for hydride transfer from NADH with turnover numbers (TONs) of 8.2 and 7.6, respectively; the concentration of reacted NADH is calculated by measuring the absorption difference at 339 nm (Figure 4 b). Importantly, the ROS hydrogen peroxide (H_2_O_2_) was detected by the appearance of a blue color on an H_2_O_2_ test stick in a solution of **1**-**py** (1 mm) with NADH (3 mol equiv) in MeOH/H_2_O (3:7; Figure 4 c), revealing that H_2_O_2_ was present in a concentration of approximately 0.22 mm, the level probably being limited by the solubility of oxygen (ca. 0.23 mm at 288 K).^15^ No H_2_O_2_ was detected in the presence of added catalase or when the reaction was carried out under a nitrogen atmosphere.

**Figure 4:**
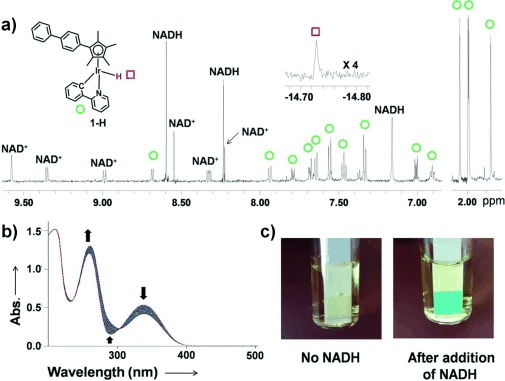
Reactions of 1-Cl and 1-py with NADH. a) ^1^H NMR spectra of the reaction between 1-Cl (0.25 mm) and NADH (3.5 mol equiv) in CD_3_OD/H_2_O (1:1) at 310 K. Left: low-field region; right: high-field region showing the resonances that arise from the Cp methyl substituents; top: Ir–H hydride peak (×4). b) UV/Vis spectra of the reaction of NADH (87 μm) with 1-Cl (0.8 μm) in MeOH/H_2_O (1.6:98.4) at 310 K for 20 h. c) Detection of hydrogen peroxide in a solution of 1-py (1 mm) with NADH (3 mol equiv) in MeOH/H_2_O (3:7, v/v) at 310 K. After 24 hours, H_2_O_2_ (ca. 0.22 mm) was detected by Quantofix peroxide test sticks.

To the best of our knowledge, hydride transfer from NADH to O_2_ has not been reported previously, although Noyori-type transfer hydrogenation catalysts, such as [(η^5^-Cp*)Ir(TsDPEN)(H)] (TsDPEN=H_2_NCHPhCHPhN(SO_2_C_6_H_4_CH_3_)^−^), can undergo oxidative addition of O_2_ to give hydroperoxide intermediates and H_2_O as a product in MeCN and CH_2_Cl_2_.^16^ The production of the ROS H_2_O_2_ by electron transfer from NADH to O_2_ might therefore be involved in the activity of **1**-**py** (and **1**-**Cl**) in cancer cells.

Electrochemical studies ruled out the possibility that an iridium-centered redox process is related to the ROS production (Figure S11).

GSH is abundant (at millimolar concentrations) in cells and participates in the detoxification of many anticancer drugs.^17^ Therefore, we investigated whether the reactions of **1**-**py** and **1**-**Cl** with GSH might be involved in the difference in their anticancer activities. ^1^H NMR spectra showed that 95 % of **1**-**py** had reacted with GSH after 12 hours to yield complex [(η^5^-Cp^xbiph^)Ir(phpy)(SG)]^−^ (**1**-**SG**; Figure S12). The four CH_3_ groups in the Cp^xbiph^ ring of **1**-**py** give rise to three singlets with an intensity ratio of 1:1:2, but split into six peaks with an intensity ratio of 1:1:2:1:1:2 for the glutathione adduct **1**-**SG** (Figure S12). Complex **1**-**py**, which contains an unsymmetric chelating ligand, is chiral; therefore, two diastereomeric glutathione adducts are expected. Other ^1^H NMR peaks for **1**-**SG** were assigned with the aid of a 2D NOESY spectrum (Figure S13). The significant upfield shifts of the resonances for Glu-*γ* CH_2_, Glu-*β* CH_2_, and Cys-*β* CH_2_ of **1**-**SG** compared to those of free GSH are notable (Figure S12 d). The formation of **1**-**SG** was confirmed by ESI-MS analysis (Figure S14). To the best of our knowledge, this is the first characterization of a cyclopentadienyl iridium complex containing glutathione as a ligand.

Whereas the substitution of pyridine in **1**-**py** by GSH was slow, the reaction of GSH with **1**-**Cl** proceeded rapidly to yield **1**-**SG** (<30 min); this difference may influence the fate of the two complexes in cells. Indeed, decreasing the cellular level of GSH with l-BSO (Figure S5) resulted in a larger increase in activity for **1**-**Cl** compared to **1**-**py**, perhaps indicating the higher extent of deactivation of **1**-**Cl** by GSH compared to the less reactive **1**-**py**.

Our studies of the aqueous chemistry of **1**-**Cl** and **1**-**py** (summarized in Figure 5) provide a molecular basis for their anticancer activity and for their differences in potency. Complex **1**-**Cl** is more reactive towards hydrolysis, GSH, and NADH than **1**-**py**. Such a high reactivity can lead to side reactions (deactivation) so that the amount of iridium species that reach intracellular target sites is reduced. The relatively unreactive complex **1**-**py** shows enhanced accumulation in cancer cells, which is followed by the reaction with NADH and the generation of the ROS hydrogen peroxide. In cells, this also appears to lead to a build-up of superoxide. The higher level of iridium accumulation in A2780 ovarian cancer cells after treatment with **1**-**py** is consistent with its ability to generate higher levels of ROS compared to **1**-**Cl** and its higher anticancer potency.

**Figure 5:**
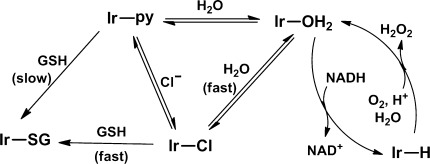
Possible reaction pathways for the production of H_2_O_2_. Replacement of chloride in 1-Cl by pyridine (to give 1-py) slows down the formation of 1-SG and subsequent deactivation processes.

Herein, we have described the synthesis and characterization of the new organometallic Ir^III^ anticancer complex [(η^5^-Cp^xbiph^)Ir(phpy)(py)]^+^ (**1**-**py**). The presence of the strongly bound pyridine ligand slows down reactions (such as hydrolysis) by several orders of magnitude compared to those of its chlorido analogue **1**-**Cl**. The glutathione adduct **1**-**SG** is formed much more slowly from complex **1**-**py** than from **1**-**Cl**, leading to less deactivation. Complex **1**-**py** was found to exhibit nanomolar activity in a wide range of cancer cell lines in the NCI-60 screen, and is therefore an order of magnitude more potent than the anticancer drug cisplatin. In comparison to **1**-**Cl**, **1**-**py** has a more promising therapeutic index towards cancer cells compared to normal cells.

Importantly, the iridium complexes have a MoA that is different from that of platinum drugs. Remarkably, **1**-**py** induces a significant increase in the level of ROS in ovarian cancer cells within one hour and is the first reported organometallic iridium compound to do so. As would be expected for an oxidant drug, the activity of **1**-**py** is potentiated by l-BSO. Complex **1**-**py** accumulates in cancer cells to a greater extent than **1**-**Cl** and generates higher levels of ROS.

The potential use of synthetic metal complexes for catalyzing chemical transformations in living organisms is currently attracting much attention.^18^ Our chemical studies reveal a basis for a novel oxidant MoA of **1**-**py** and **1**-**Cl**, which involves catalytic hydride transfer from the coenzyme NADH to oxygen to produce the ROS H_2_O_2_ as a product. This new strategy for the rational design of oxidant catalytic organoiridium drugs may be highly effective for treating platinum-resistant cancers.

## Supporting Information

As a service to our authors and readers, this journal provides supporting information supplied by the authors. Such materials are peer reviewed and may be re-organized for online delivery, but are not copy-edited or typeset. Technical support issues arising from supporting information (other than missing files) should be addressed to the authors.

miscellaneous_information
