# Supplementary material for: The Potent Oxidant Anticancer Activity of Organoiridium Catalysts
Source: Angew Chem Int Ed Engl. 2014 Mar 11;53(15):3941–6. doi: 10.1002/anie.201311161 (PMC4227564; doi:10.1002/anie.201311161)
Supplement: Supplementary file 1 — miscellaneous_information [file anie0053-3941-SD1.pdf]

Angewandte Chemie
Eine Zeitschrift der Gesellschaft Deutscher Chemiker

Supporting Information

© Wiley-VCH 2014

69451 Weinheim, Germany

The Potent Oxidant Anticancer Activity of Organoiridium Catalysts**

*Zhe Liu, Isolda Romero-Canelón, Bushra Qamar, Jessica M. Hearn, Abraha Habtemariam, Nicolas P. E. Barry, Ana M. Pizarro, Guy J. Clarkson, and Peter J. Sadler**

anie_201311161_sm_miscellaneous_information.pdf

Supporting Information

Materials	S2-S3
Methods and Instrumentation	S3-S11
Tables S1-S4	S12-S15
Figures S1-S14	S16-S24
References	S25

Materials. 2-Phenylpyridine, pyridine, ammonium hexafluorophosphate, silver nitrate, 9-ethylguanine (9-EtG), 9-methyladenine (9-MeA), L-buthionine sulfoximine (L-BSO), H_2O_2 (30%), quantofix peroxides test sticks, catalase from bovine liver (aqueous suspension), *β*-nicotinamide adenine dinucleotide reduced dipotassium salt (NADH), and L-glutathione reduced (GSH) were purchased from Sigma-Aldrich. ROS/superoxide detection kit was purchased from Enzo-Life sciences.

[(η^5-Cp^{xbiph})Ir(phpy)Cl] (**1-Cl**) was synthesized as described previously.[1]

Preparation of [(η^5-Cp^{xbiph})Ir(phpy)(py)]PF_6 (1-py·PF_6). A solution of [(η^5-Cp^{xbiph})Ir(phpy)Cl] (**1-Cl**) (33 mg, 0.05 mmol) and $AgNO_3$ (8.5 mg, 0.05 mmol) in MeOH (10 mL) and water (20 mL) was stirred at ambient temperature for 24 h. The precipitate (AgCl) was removed by filtration through a glass wool plug, and pyridine (40 mg, 0.50 mmol) was added to the filtrate. The reaction mixture was stirred at ambient temperature for 12 h. The volume was slowly reduced to half on a rotary evaporator and NH_4PF_6 (45 mg, 0.28 mmol) was added. The yellow precipitate that formed was collected by filtration, washed with diethyl ether, and recrystallized from methanol/diethyl ether. Yield: 27 mg (32%). ^{1}H NMR($CDCl_3$): δ = 8.97 (d, 1H, *J* = 5.8 Hz), 8.50 (d, 2H, *J* = 6.0 Hz), 7.76 (m, 3H), 7.66 (m, 2H), 7.51 (d, 2H, *J* = 7.2 Hz), 7.41 (m, 5H), 7.33 (m, 4H), 7.18 (t, 1H, *J* = 7.6 Hz), 6.87 (d, 2H, *J* = 7.6 Hz), 1.86 (s, 3H), 1.78 (s, 3H), 1.62 (s, 3H), 1.57 (s, 3H). ^{13}C NMR(MeOD-d_4): δ = 154.66, 153.02, 140.49, 136.44, 133.44, 131.84, 130.00, 128.77, 128.27, 127.88, 125.98, 125.43, 50.77. Anal. Calcd for $C_{37}H_{34}F_6IrN_2P$ (843.86): C, 52.66; H, 4.06; N, 3.32. Found: C, 52.86; H, 3.99; N, 3.40. MS: *m/z* 699.1 [M – PF_6]$^+$. Crystals suitable for X-ray diffraction were obtained by slow diffusion of diethylether into a methanol/acetonitrile solution of the complex at ambient temperature.

X-ray crystal structure of 1-py. X-ray crystallographic data for [(η^5-Cp^{xbiph})Ir(phpy)py]PF_6·$(CH_3OH)_{0.5}$ are shown in Table S1, and selected bond lengths and angles listed in Table S2. There are only two previous reports of crystal

structures of complexes containing the Cp^{xbiph} ligand.[1-2] The complex adopts the expected half-sandwich pseudo-octahedral ‘three-leg piano-stool’ geometry with the Ir bound to a η^5-cyclopentadienyl ligand occupying 3 coordination sites, a nitrogen atom from the py ligand and a *C^N*-chelated phpy ligand. The distance from Ir to the centroid of cyclopentadienyl ring is 1.838 Å, slightly longer than that of **1-Cl** (1.825 Å).[1] The central ring of Cp^{xbiph} is twisted about 86.2° relative to the cyclopentadienyl ring, while the angle between mean planes through the terminal phenyl ring and cyclopentadienyl ring is 39.8°. The twist angle between the phpy ligand and py ligand is 72.2°. The Ir-C(phpy) has similar bond length to that of Ir-N(phpy). However, the Ir-N(phpy) bond length [2.068(5) Å] is significantly shorter than that of Ir-N(py) [2.114(5)Å].

Methods and Instrumentation.

NMR Spectroscopy. ^{1}H NMR spectra were acquired in 5 mm NMR tubes at 298 K or 310 K on either a Bruker DPX 400 (^{1}H = 400.03 MHz) or AVA 600 (^{1}H = 600.13 MHz) spectrometers. ^{1}H NMR chemical shifts were internally referenced to $C\mathit{H}D_2OD$ (3.33 ppm) for methanol-d_4 or $C\mathit{H}Cl_3$ (7.26 ppm) for chloroform-d_1. MeOD-d_4 was used to aid solubility. 2D ^{1}H–^{1}H TOCSY NMR spectrum for compound **1-py** and ^{1}H–^{1}H NOESY NMR spectrum for reaction of **1-py** with glutathione were recorded at 298 K. All data processing was carried out using MestReC or TOPSPIN version 2.0 (Bruker U.K. Ltd.).

Electrospray Ionization Mass Spectrometry (ESI-MS). Electrospray ionization mass spectra were obtained by preparing the samples in 50% CH_3CN and 50% H_2O (v/v) or using NMR samples for infusion into the mass spectrometer (Bruker Esquire 2000). The mass spectra were recorded with a scan range of *m/z* 400–1000 for positive ions.

Elemental Analysis. CHN elemental analyses were carried out on a CE-440 elemental analyzer by Warwick Analytical (UK) Ltd.

High Pressure Liquid Chromatography (HPLC). Reverse phase HPLC (RP-HPLC) was performed on a HP 1100 Series HPLC System (Agilent) using a ZORBAX Eclipse Plus C-18 column. The mobile phases were A: water (HPLC grade, Aldrich) containing 0.1% trifluoroacetic acid (TFA) and B: methanol (HPLC grade, Aldrich) containing 0.1% TFA. A flow rate of 1 mL min^{-1} was used.

X-ray Crystallography. A suitable crystal was selected and mounted on a glass fibre with Fromblin oil on an Oxford Diffraction Gemini Xcalibur diffractometer with a Ruby CCD area detector. The crystal was kept at 150(2) K during data collection. Using Olex2.[3] the structure of compound **1** was solved with the XS structure solution program[4] using Direct Methods, and refined with the XL refinement package using Least Squares minimization.[4]

X-ray crystallographic data for compound **1-py** have been deposited in the Cambridge Crystallographic Data Centre under the accession number CCDC 956067.

pH Measurement. pH or pH* (pH meter reading without correction for effects of deuterium on glass electrode) values of NMR samples in H_2O or D_2O were measured at ca. 298 K directly in the NMR tube, before and after recording NMR spectra, using a Corning 240 pH meter equipped with a micro combination electrode calibrated with Aldrich buffer solutions of pH 4, 7 and 10.

UV-Vis Spectroscopy. A Cary 300 UV-Vis recording spectrophotometer was used with 1 cm path-length quartz cuvettes (0.5 mL) and a PTP1 Peltier temperature controller. Spectra were processed using UVWinlab software. Experiments were carried out at 310 K from 600 to 200 nm.

Inductively Coupled Plasma-Mass Spectrometry (ICP-MS). All ICP-MS analyses were carried out on an Agilent Technologies 7500 series ICP-MS instrument. The

water used for ICP-MS analysis was doubly deionized (DDW) using a Millipore Milli-Q water purification system and a USF Elga UHQ water deionizer. The iridium Specpure plasma standard (Alfa Aesar, 1000 ppm in 10% HCl) was diluted with 5% HNO_3 DDW to prepare freshly calibrants at concentrations of 50000, 10000, 5000, 1000, 500, 200, 50, 10, and 5 ppt. The ICP-MS instrument was set to detect ^{193}Ir with typical detection limits of ca. 2 ppt using no-gas mode.

Electrochemistry. All cyclic voltammogram (CV) experiments were carried out using a CH Instrument model 600D Electrochemical Analyzer/Workstation (Austin, TX). The electrochemical measurements were performed on complex **1-py** (1.0 mM) in a 70/30 (V/V) methanol/water solution containing tetrabutylammonium hexafluorophosphate (0.1 M) as supporting electrolyte. The solutions were degassed under nitrogen and cyclic voltammograms were scanned from -2.0 V to +0.8 V (three complete scans for each experiment). In a typical electrochemical experimental set up, the three-electrode system was used: a glassy carbon electrode as the working electrode, Ag/AgCl in 3.0 M KCl as the reference electrode (0.21 V versus NHE), and platinum wire as the counter electrode. For each electrode, CV was performed at a scan rate of 100 mV/s.

NCI-60 Screening. Complex **1-py** was evaluated by the National Cancer Institute Developmental Therapeutics Program (NCI/DTP, U.S.A.) for *in vitro* activity towards ca. 60 human cancer cell lines. The cells were treated with iridium compounds for 48 h at 5 concentrations ranging from 0.01 to 100 μM. Every compound was tested twice and data are the average of the two experiments. Data for cisplatin and oxaliplatin are from NCI/DTP screening performed in October 2009 and 2010, respectively. The protocol for the determination of cytotoxicity on the 60 cell line panel can be found at http://dtp.nci.nih.gov/branches/btb/ivclsp.html. The DTP homepage can be accessed at http://dtp.cancer.gov/.

Cell Culture. A2780 human ovarian carcinoma cells were obtained from the European Collection of Cell Cultures (ECACC) and used between passages 5 and 18. MRC-5 human fetal lung fibroblasts were also obtained from the same source. Both cell lines were grown in Roswell Park Memorial Institute medium (RPMI-1640) supplemented with 10% of fetal calf serum, 1% of 2 mM glutamine and 1% penicillin/streptomycin. They were grown as adherent monolayers at 310 K in a 5% CO_2 humidified atmosphere and passaged at approximately 70-80% confluence.

***In vitro* Growth Inhibition Assay.** Briefly, 5000 cells were seeded per well in 96-well plates. The cells were pre-incubated in drug-free media at 310 K for 48 h before adding different concentrations of test compounds. Stock solutions of the Ir^{III} complexes were firstly prepared in 5% DMSO (v/v) and a mixture 0.9% saline and RPMI-1640 medium (1:1) (v/v) following serial dilutions in RPMI-1640.The drug exposure period was 24 h. After this, supernatants were removed by suction and each well was washed with PBS. The cells were allowed to recover for a further 72 h in drug-free medium at 310 K. The SRB assay was used to determine cell viability.[5] Absorbance measurements of the solubilised dye (on a BioRad iMark microplate reader using a 470 nm filter) allowed the determination of viable treated cells compared to untreated controls. IC_{50} values were determined as duplicates of triplicates in two independent sets of experiments and their standard deviations were calculated.

Time-dependence of *In vitro* Growth Inhibition. These experiments were carried out for complex **1-py**, as described above (*In vitro* Growth Inhibition Assay) with the following experimental modifications: variable exposure times were 0.5, 1, 2 and 4 h.

Modulation of IC_{50} for Complex 1-py on Depletion of GSH. A 96-well plate was seeded with 5000 A2780 ovarian cells per well. Cells were pre-incubated in drug-free medium for 48 h at 310 K, before adding **1-py** together with L-BSO. In order to prepare a stock solution of the drug, the solid complex was dissolved in 5% DMSO

(v/v) and a mixture 0.9% saline and medium (1:1 v/v). This stock was further diluted using RPMI-1640 until working concentrations were achieved. Separately, a stock solution of L-BSO was prepared in saline. Both solutions were added to each well independently, but within 5 min of each other. After 24 of exposure, drugs were removed by suction, cells were washed with PBS and fresh medium was added to the plate. Cells were allowed to recover in drug-free medium for 72 h at 310 K. At the end of this period, the SRB assay was used to determine cell viability. These experiments included two negative controls: untreated cells and cells exposed to 5 μM of L-BSO. Under our experimental conditions, L-BSO has an IC_{50} >200 μM and thus deemed non-toxic. In all cases, the differences between the two negative controls were not statistically significant (<2%). This confirmed that the concentration of L-BSO used was non-toxic. IC_{50} values were determined as duplicates of triplicates in two independent sets of experiments and their standard deviations were calculated.

ROS Determination. Flow cytometry analysis of ROS/superoxide generation in A2780 and MRC-5 cells caused by exposure to complexes **1-py** and **1-Cl**, was carried out using the Total ROS/Superoxide detection kit (Enzo-Life sciences) according to the supplier's instructions. Briefly, 1.5×10^6 A2780 cells per well were seeded in a 6-well plate. Cells were pre-incubated in drug-free media at 310 K for 24 h in a 5% CO_2 humidified atmosphere, then **1-py** was added to triplicates at concentrations of 1/3 x IC_{50}, IC_{50} and 3 x IC_{50}. After 1 h of drug exposure, supernatants were removed by suction and cells were washed with PBS. Cells were harvested using trypsin and collected after centrifugation. This experiment included pyocyanin as a positive control and two negative controls, the first using untreated cells and the second cells incubated with NAC. There were no statistically significant differences between the two negative controls. Staining was achieved by re-suspending the cell pellets in buffer containing the orange/green fluorescent reagents. Cells were analysed on a Becton Dickinson FACScan Flow Cytometer using FL1 channel Ex/Em: 490/525 nm

for the oxidative stress and FL2 channel Ex/Em: 550/620 nm for superoxide detection. Compensation adjustments were carried out using pyocyanin-treated cells singly-stained with either fluorescent agent. Data were processed using Flowjo software.

ROS Inhibition by N-acetyl-L-cysteine (NAC). Flow cytometry analysis of A2780 cells after co-administration of **1-py** or **1-Cl** and NAC was carried out as described above with the following modification. Separate stock solutions of **1-py** or **1-Cl** at concentrations of 1/3 x IC_{50}, IC_{50} and 3 x IC_{50} and 5 µM NAC were prepared in cell culture medium. Both solutions were added to each well independently, but within 5 min of each other. Drug exposure time was 1 h. These experiments included two negative controls: untreated cells, and cells treated only with NAC. In both cases the majority of the population appears in the lower left quadrant of an FL2 vs FL1 dot plot. There were no statistically significant differences between both negative controls.

Effect of NAC on superoxide production. Flow cytometry studies to confirm the limited ability of NAC to suppress the induction of superoxide species were carried out as described above with the following modification. Cells were exposed to 1 µM pyocyanin and 5 µM NAC for 1 h.

We studied the effect of co-administering the ROS scavenger NAC with **1-py** to ovarian cancer cells. A2780 cells were treated with 3 × IC_{50} of **1-py** and 5 µM NAC for 1 h. A drastic reduction in green fluorescence was observed in the FL-1 channel (Figure 3B), indicating a marked decrease in the total ROS level. However, superoxide levels remained unchanged, suggesting that even in the presence of a thiol such as NAC, the Ir complex is able to increase the level of superoxide in A2780 cancer cells. Similar results were achieved with 1/3 × IC_{50} and IC_{50} concentrations of **1-py**. The corresponding experiments with **1-Cl** showed a consistent reduction of total ROS levels, with lower superoxide levels compared to **1-py** (65.8 % vs 97.5 %

population in the –FL-1 /+FL-2 quadrant). The limited ability of NAC to inhibit superoxide production has been reported before.[6] This was confirmed by incubating A2780 cells with a positive control (pyocyanin) and NAC, for which the ROS level is reduced, but the superoxide level remains unchanged (95 % of the cell population moved from +FL-1/+FL-2 only with pyocyanin to the –FL1/+FL2 quadrant after adding NAC).

Iridium Accumulation in Cancer Cells. Cell accumulation studies for complexes **1-Cl** and **1-py**, were conducted on A2780 ovarian cells. Briefly, 4 x 10^6 cells were seeded on a Petri dish. After 24 h of pre-incubation time in drug-free medium at 310 K, the complexes were added to give final concentrations equal to IC_{50}/3 and a further 24 h of drug exposure was allowed. After this time, cells were treated with trypsin, counted, and cell pellets were collected. Each pellet was digested overnight in concentrated nitric acid (73%) at 353 K; the resulting solutions were diluted with double-distilled water to a final concentration of 5% HNO_3 and the amount of Ir taken up by the cells was determined by ICP-MS. These experiments did not include any cell recovery time in drug-free media; they were carried out in duplicate of triplicates in two independent experiments and the standard deviations were calculated.

Time- and Concentration- Dependence of Cellular Accumulation. These experiments were carried out for complex **1-py** in A2780 ovarian cells, as described above with the following experimental modifications: variable exposure times were 0.5, 1, 2 and 4 h. Concentrations were equal to 1/3 x IC_{50}, IC_{50} and 3 x IC_{50}.

Hydrolysis. Solutions of complex **1-py** with final concentrations of 0.1 mM in 10% MeOD-d_4/90% D_2O (v/v) were prepared by dissolution of the complex in MeOD-d_4 followed by rapid dilution with D_2O. ^1H NMR spectra were recorded after various time intervals at 310 K.

Stability of 1-py in NaCl solution. Different amount of NaCl was added to an NMR tube containing a 0.7 mM solution of complex **1-py** in 30% MeOD-d_4/70% D_2O at ambient temperature to prepare a solution containing 104, 23, or 4 mM NaCl. ^{1}H NMR spectra of the resulting solutions were recorded at 310 K and various time intervals for 24 h.

Interactions of 1-Cl with NADH. NADH (3.5 mol equiv) was added to an NMR tube containing a 0.25 mM solution of complex **1-Cl** in 50% MeOD-d_4/50% H_2O at ambient temperature. ^{1}H NMR spectra of the resulting solutions were recorded at 310 K and various time intervals for 24 h.

Interactions of 1-py with NADH. NADH (2.5 mol equiv) was added to an NMR tube containing a 0.8 mM solution of complex **1-py** in 30% MeOD-d_4/70% H_2O at ambient temperature. ^{1}H NMR spectra of the resulting solutions were recorded at 310 K and various time intervals for 24 h.

UV-Vis detected catalytic reaction of 1-py and 1-Cl with NADH. Reaction between **1-py** (0.8 μM) and **1-Cl** (0.8 μM) with NADH (87 μM) in 1.6% MeOH/98.4% H_2O was monitored by UV-Vis at 310 K for 20 h. Turnover number (TON) is defined as the number of moles of NADH that a mole of catalyst (**1-py** or **1-Cl**) can convert within 20 h. TON was calculated from the difference in NADH concentration after 20 h divided by the concentration of iridium catalyst. The concentration of NADH was obtained using the extinction coefficient ε_{339} = 6220 $M^{-1}cm^{-1}$.

Detection of H_2O_2. For the reaction of **1-py** (1 mM) with 3 mol equiv NADH in 30% MeOH/70% H_2O (v/v) at 310 K, H_2O_2 was detected by quantofix peroxide test sticks.

Interaction of 1-py with GSH. GSH (3.5 mol equiv) was added to an NMR tube containing a 2 mM solution of complex **1-py** in 30% MeOD-d_4/70% phosphate buffer (H_2O) at ambient temperature, pH = 7.4. ^{1}H NMR spectra of the resulting solutions were recorded at 310 K and various time intervals for 24 h.

Interactions of 1-Cl with GSH. GSH (3.5 mol equiv) was added to an NMR tube containing a 0.25 mM solution of complex **1-Cl** in 50% MeOD-d_4/50% PBS H_2O buffer (pH = 7.4) at ambient temperature. ^{1}H NMR spectra of the resulting solutions were recorded at 310 K and various time intervals for 24 h.

Electrochemical studies. To rule out the possibility that the metal-centered redox process Ir^{III}/Ir^{IV} was responsible for the ROS production, the electrochemical behavior of complex **1-py** was studied by cyclic voltammetry (CV). The cyclic voltammogram showed two well-separated irreversible electrochemical waves (Figure S10A), a reduction wave at -1.60 V and an oxidation wave at +0.05 V. However, the corresponding anodic and cathodic peaks in the reverse scan are absent, attributable to the instability of species formed.[7] The oxidation is likely to be assignable to an Ir^{III}/Ir^{IV} couple,[7b] while the irreversible reduction may be attributable to ligand-centered reduction of 2-phenylpyridine.[7a] Interestingly, ligand reduction needs to occur prior to oxidation of Ir^{III} to Ir^{IV} (Figure S10B). Since the irreversible ligand-centered reduction wave at -1.60 V is too negative to be biologically accessible, the production of ROS is unlikely to be related to Ir^{III}/Ir^{IV} redox process.

Table S1. Crystallographic data for $[(\eta^5\text{-Cp}^{xbiph})Ir(phpy)py]PF_6\cdot(CH_3OH)_{0.5}$.

formula	$C_{37.5}H_{36}F_6IrN_2PO_{0.5}$
MW	859.85
cryst. size (mm)	0.20× 0.20 × 0.12
λ (Å)	0.71073
temp(K)	150
Z	8
cryst. syst.	orthorhombic
space group	Pbcn
a (Å)	14.3401(4)
b (Å)	16.5924(3)
c (Å)	28.5886(8)
α (°)	90
β (°)	90
γ (°)	90
vol(Å^3)	6802.3(3)
abs. coeff. (mm^{-1})	4.037
F(000)	3400.0
2θ range for data collection (deg)	5.85 to 63.752
index ranges	$-20 \le h \le 19$, $-22 \le k \le 24$, $-39 \le l \le 41$
reflections collected	43018
independent reflections	10758 [*R*(int) = 0.0561]
data/restraints/params	10758/90/434
final *R* indices [$I > 2\sigma(I)$]	R_1 = 0.0732, wR_2 = 0.1123
largest diff. peak/hole (e Å^{-3})	2.40 /-1.64
GOF	1.133

Table S2. Selected bond lengths (Å) and angles (deg) for $[(\eta^5\text{-Cp}^{xbiph})\text{Ir(phpy)py}]\text{PF}_6\cdot(\text{CH}_3\text{OH})_{0.5}$.

Bond/Angle [a]	Å/deg
Ir−C31	2.167(7)
Ir−C32	2.203(7)
Ir−C33	2.210(7)
Ir−C34	2.206(7)
Ir−C35	2.221(9)
Ir−C(centroid)	1.838
Ir−C12	2.070(5)
Ir−N1	2.068(5)
Ir−N13	2.114(5)
C12−Ir−N1	77.9(2)
C12−Ir− N13	89.63(18)
N1−Ir− N13	88.76(18)

[a] See Figure 1 for atom labelling

Table S3. Antiproliferative activity of complexes **1-Cl**, **1-py** and CDDP (cisplatin) towards A2780 human ovarian cancer cells and MRC-5 normal human fibroblasts, iridium accumulation in A2780 cells, and effect of co-treatment with the redox modulator L-BSO.

	A2780			MRC-5
Complex	IC_{50} (µM)[a]	+ L-BSO IC_{50} (µM)[b]	Cellular Ir accumulation (ng Ir/10^6 cells)[c]	IC_{50} (µM)[d]
**1-Cl**	0.70 ± 0.04	0.11 ± 0.01	0.39 ± 0.05	2.71 ± 0.01
**1-py**	0.12 ± 0.02	0.060 ± 0.003	8.3 ± 0.3	1.6 ± 0.3
**CDDP**	1.2 ± 0.1	nd	nd	16.2 ± 0.6

[a] 48 h of pre-incubation, 24 h of drug exposure and 72 h of cell recovery in drug-free medium.

[b] Complexes co-administered with 5 µM of L-BSO. Under these conditions, the IC_{50} for L-BSO is > 200 µM. The differences in cell viability between cells treated with 5 µM of L-BSO and the untreated control were all < 2%.

[c] Ir accumulation in A2780 cells following 24 h pre incubation and 24 h drug exposure. The concentrations used were equipotent (1/3 × IC_{50}). No recovery time in drug-free medium.

[d] Values determined following 48 h of pre-incubation, 24 h of drug exposure, and 72 h of cell recovery in drug-free medium.

Table S4. Time dependence of the antiproliferative activity and cellular accumulation of Ir for complex **1-py** in A2780 ovarian cancer cells.

Exposure time[a]	Cellular accumulation (ng Ir/10^6 cells)[b]			IC_{50} (µM)
	1/3 × IC_{50}	IC_{50}	3 × IC_{50}	
30 min	0.47 ± 0.05	1.54 ± 0.08	4.78 ± 0.09	0.57 ± 0.03
1 h	0.65 ± 0.03	1.91 ±0.07	5.1 ± 0.1	0.18 ± 0.02
2 h	0.72 ± 0.05	2.15 ± 0.09	6.4 ± 0.2	0.141 ± 0.005
4 h	0.86 ± 0.04	3.2 ± 0.2	7.4 ± 0.1	0.13 ± 0.03

[a] 48 h of pre-incubation time, variable of drug exposure periods (30 min, 1, 2 and 4 h) and 72 h of cell recovery time in drug-free medium.

[b] 24 h of pre-incubation time and variable drug exposure periods at equipotent concentrations (1/3 × IC_{50}, IC_{50} and 3 × IC_{50}). No cell recovery time in drug-free medium.

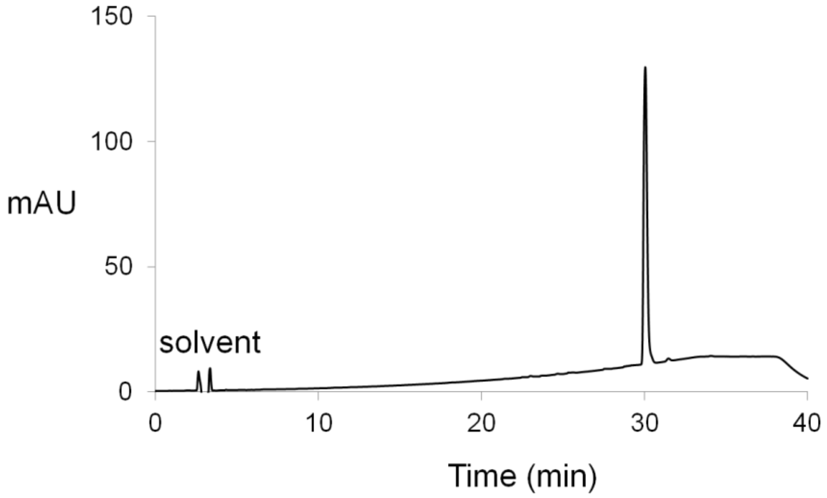

Figure S1. HPLC trace for complex **1-py**. Elution gradient: 0 – 30 min, mobile phase B: 25% – 80%; 31 – 35 min, B: 80%; 36 – 40 min, B: 80% – 25%. The mobile phases were A: water containing 0.1% TFA, and B: methanol containing 0.1% TFA. A flow rate of 1 mL min^{-1} was used. The purity of **1-py** from this trace is 98.6%.

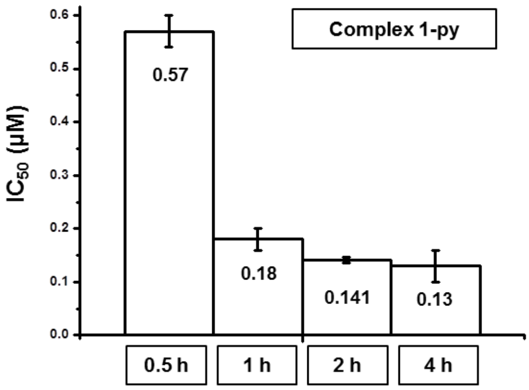

Figure S2. Time-dependence of the IC_{50} of **1-py** in A2780 cells: 48 h pre-incubation, variable drug exposure (0.5, 1, 2 and 4 h) and 72 h of recovery in drug-free medium.

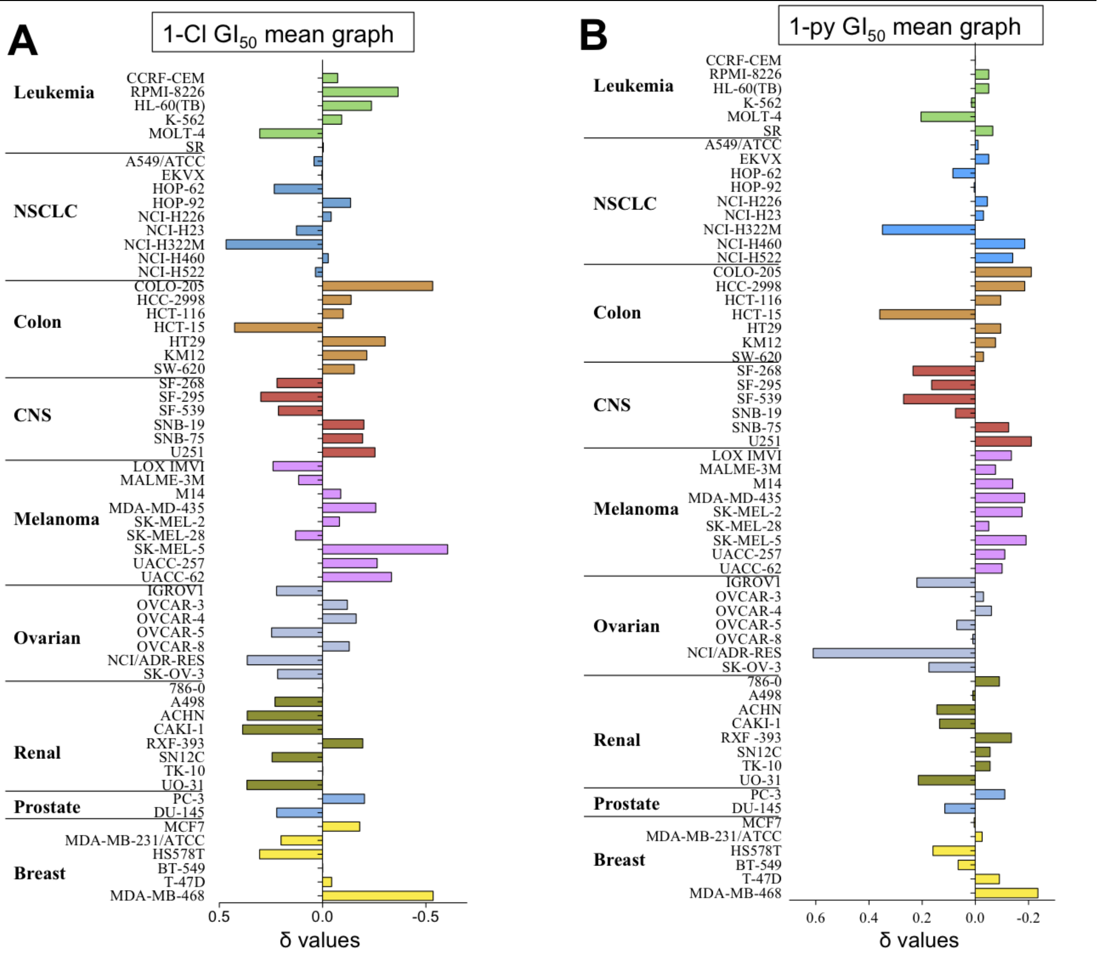

Figure S3. (A) NCI-60 GI_{50} mean graph for **1-Cl**. The midpoint ($\log_{10} GI_{50}$) is -6.15.[8] (B) NCI-60 GI_{50} mean graph for **1-py**. The midpoint ($\log_{10} GI_{50}$) is -6.63. Projections to the right indicate cell lines with susceptibility that exceeds the mean, projections to the left indicate cell lines with lower susceptibility than the mean.

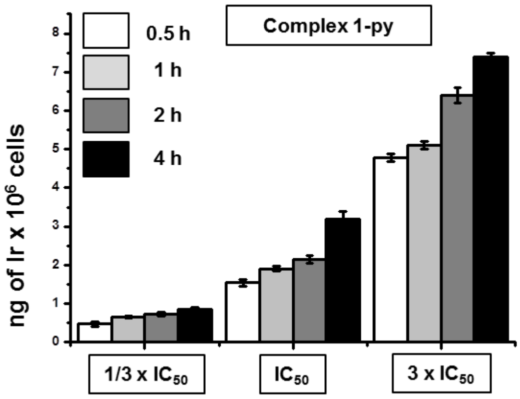

Figure S4. Accumulation of Ir from **1-py** in A2780 cells: 24 h pre-incubation and variable drug exposure (0.5, 1, 2 and 4 h), at 1/3 × IC_{50}, IC_{50} and 3 × IC_{50}, no recovery time.

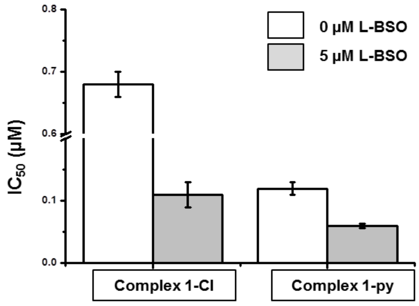

Figure S5. IC_{50} modulation experiments for **1-Cl**[8] and **1-py** in A2780 cells by depletion of GSH using 5 µM L-BSO.

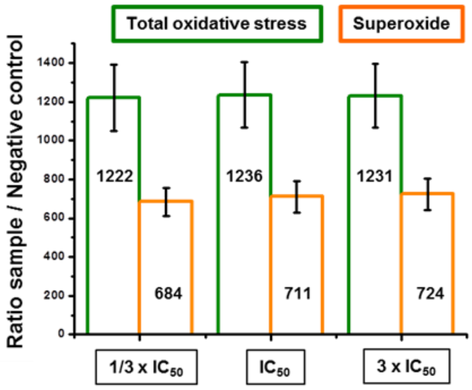

Figure S6. Concentration-dependence of induction of ROS in A2780 cells: using 1/3 × IC_{50}, IC_{50} and 3 × IC_{50} of **1-py**.

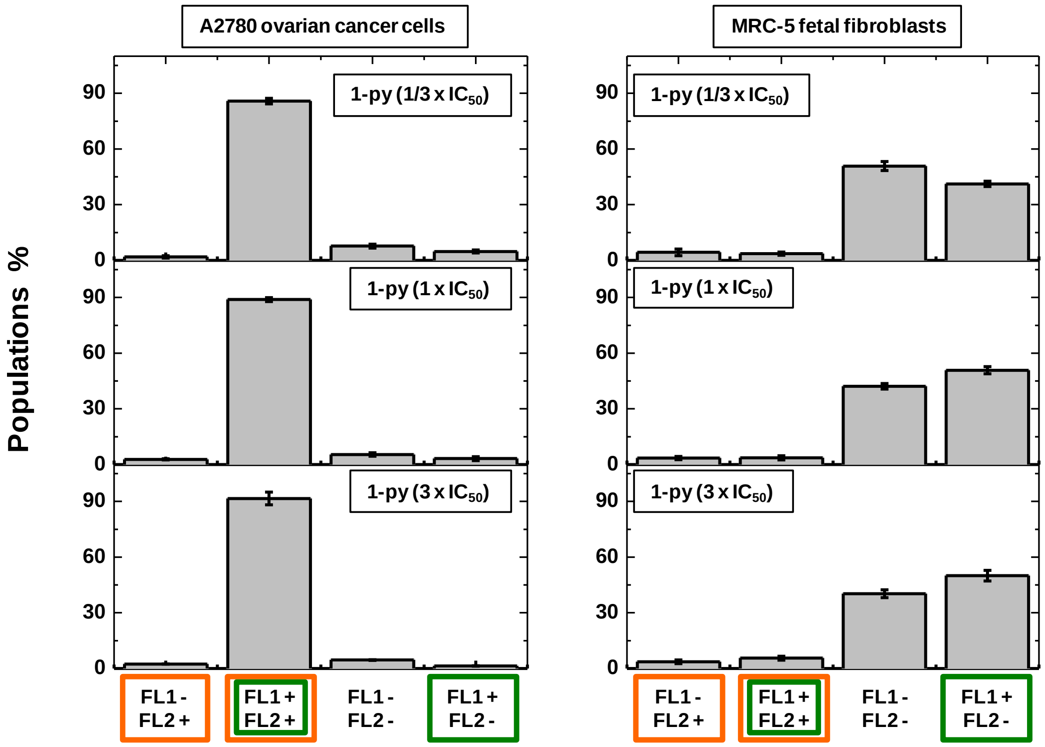

Figure S7. Comparison between the four different populations from the dot plots of ROS induction measurements by flow cytometry in A2780 ovarian cancer cells and MRC-5 fetal fibroblasts: 1 h drug-exposure at 310 K for cells treated with 1/3 x IC_{50}, IC_{50} and 3 x IC_{50} of **1-py**. FL1-green channel detects total oxidative stress and FL2-orange channel detects superoxide production.

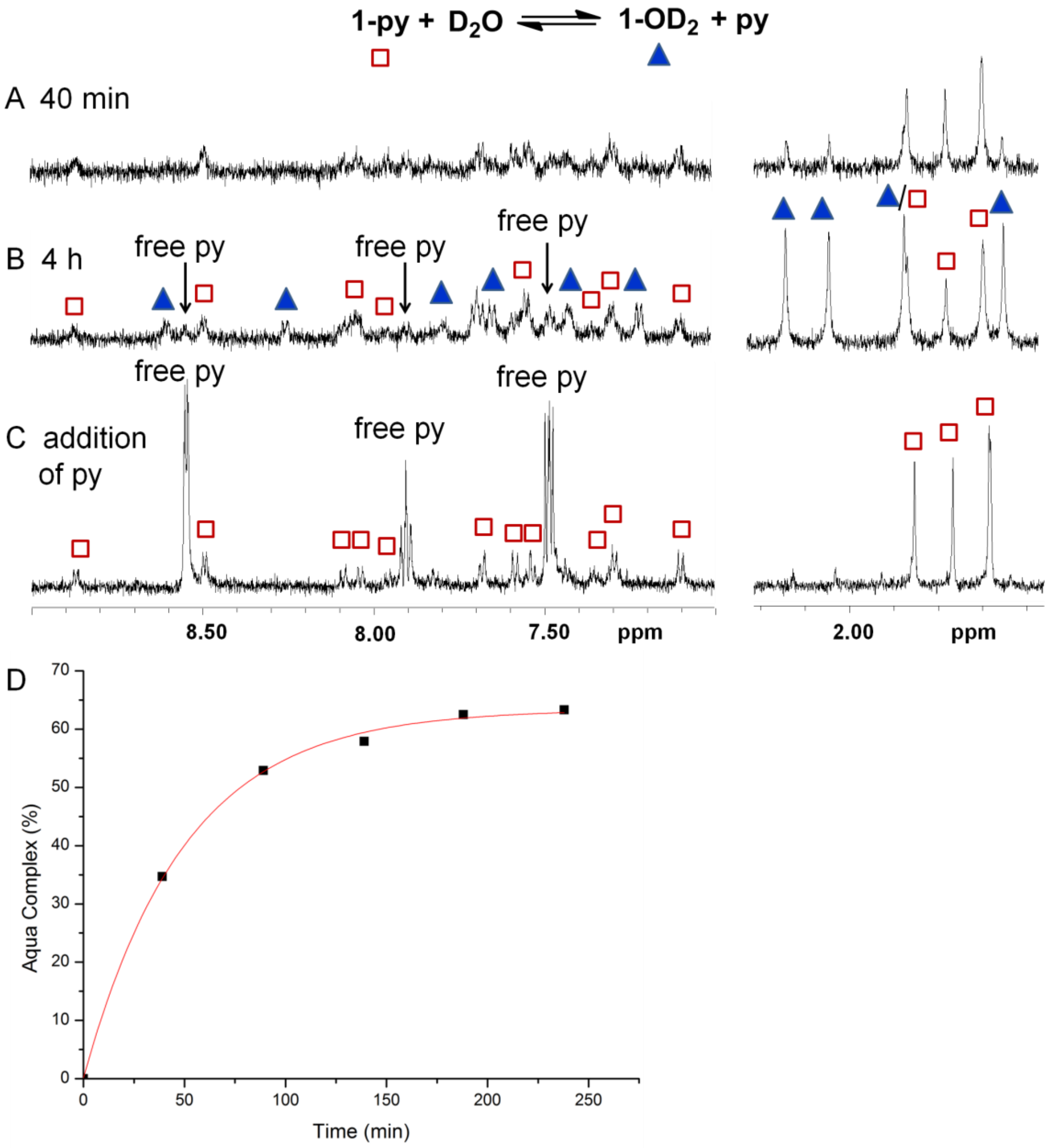

Figure S8. ^{1}H NMR spectra showing hydrolysis of complex **1-py** (100 μM) in 10% MeOD-d_4/90% D_2O at 310 K. (A) After 40 min; (B) after 4 h, when equilibrium was reached. (C) Addition of pyridine (1.1 mM) reversed the hydrolysis. (D) Time dependence for formation of aqua complex.

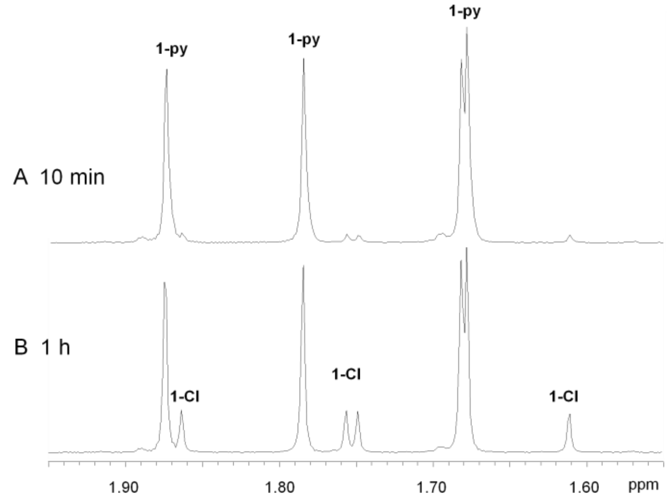

Figure S9. ^{1}H NMR spectrum showing the conversion of complex **1-py** (0.7 mM) into **1-Cl** in the presence of 104 mM NaCl in 30% MeOD-d_4/70% D_2O at 310 K. (A) 10 min; (B) after 1 h, 18% of **1-py** was converted to **1-Cl**.

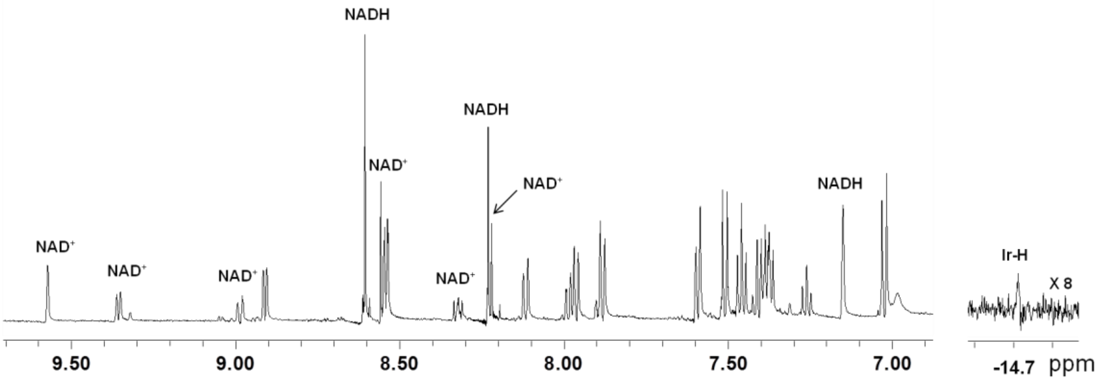

Figure S10. ^{1}H NMR spectra for reaction between **1-py** (0.8 mM) and 2.5 mol equiv of NADH in 30% MeOD-d_4/70% H_2O at 310 K. Left: low-field region; right: Ir-H hydride peak (×8).

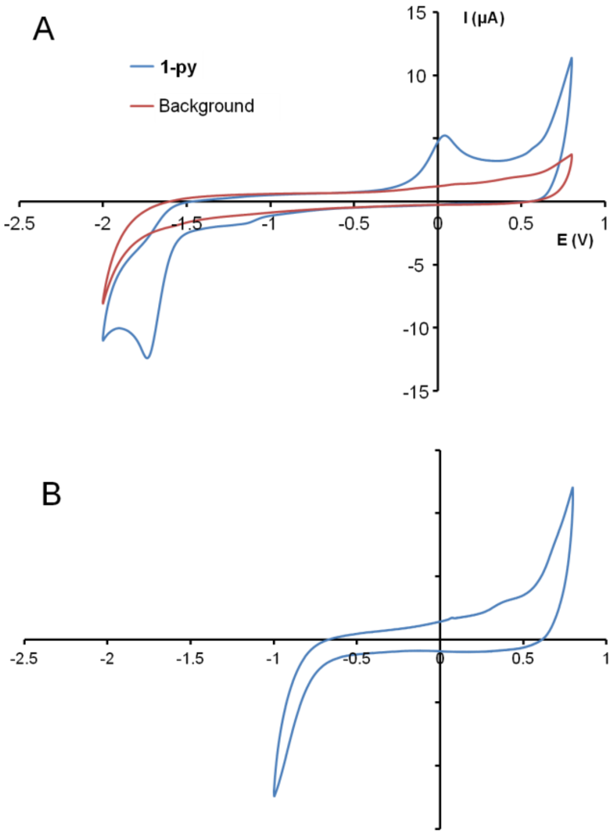

Figure S11. (A) Cyclic voltammogram of complex **1-py** (1 mM) in 70% MeOH/30% H_2O (v/v, degassed under N_2) with 0.1 M NBu_4PF_6 as supporting electrolyte. Scan rate 100 mV/s. (B) Cyclic voltammogram of complex **1-py** showing that the reduction process has to take place for observing the later oxidation.

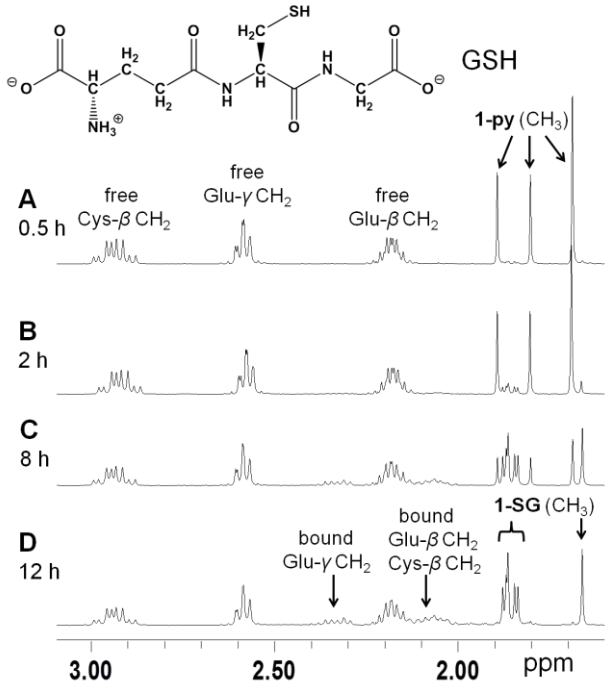

Figure S12. Formation of **1-SG**. The high field region of ^{1}H NMR spectra for reaction of **1-py** (2 mM) with GSH (7 mM) in 30% MeOD-d_4/70% phosphate buffer (H_2O) at 310 K, pH = 7.4. (A) 0.5 h; (B) 2 h; (C) 8 h; (D) after 12 h, 95% **1-py** had reacted and the product [(η^5-Cp^{xbiph})Ir(phpy)(SG)]$^-$ (**1-SG**) had formed.

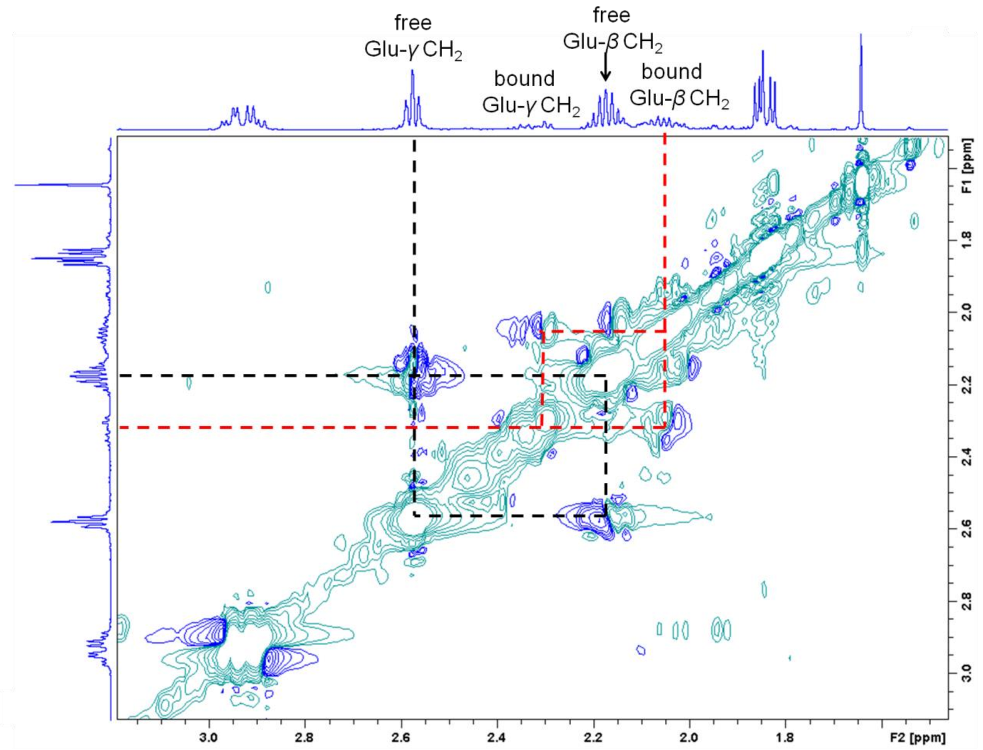

Figure S13. 2D NOESY ^{1}H NMR spectrum for an equilibrium solution of **1-py** (7 mM) and GSH (22 mM) in 50% MeOD-d_4/50% PBS D_2O buffer (v/v) at 298 K.

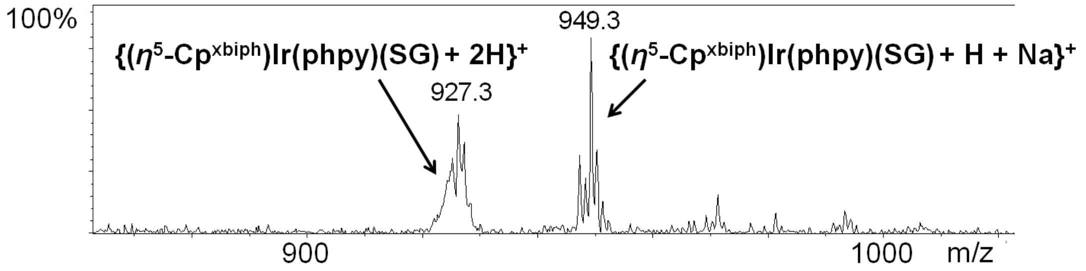

Figure S14. ESI-MS for an equilibrium solution of GSH (0.7 mM) and **1-py** (0.2 mM) in 50% CH_3CN/50% H_2O (v/v), 298 K. The peak at *m/z* 927.3 is assignable to the glutathione complex $\{(\eta^5\text{-Cp}^{xbiph})\text{Ir(phpy)(SG)} + 2\text{H}\}^+$ (calcd *m/z* 927.2).

References

[1] Z. Liu, A. Habtemariam, A. M. Pizarro, G. J. Clarkson, P. J. Sadler, *Organometallics* **2011**, *30*, 4702-4710.

[2] Z. Liu, A. Habtemariam, A. M. Pizarro, S. A. Fletcher, A. Kisova, O. Vrana, L. Salassa, P. C. A. Bruijnincx, G. J. Clarkson, V. Brabec, P. J. Sadler, *J. Med. Chem.* **2011**, *54*, 3011-3026.

[3] O. V. Dolomanov, L. J. Bourhis, R. J. Gildea, J. A. K. Howard, H. Puschmann, *J. Appl. Cryst.* **2009**, *42*, 339-341.

[4] G. Sheldrick, *Acta Cryst.* **2008**, *A64*, 112-122.

[5] V. Vichai, K. Kirtikara, *Nat. Protoc.* **2006**, *1*, 1112-1116.

[6] N. Kitaoka, G. Liu, N. Masuoka, K. Yamashita, M. Manabe, H. Kodama, *Clin. Chim. Acta* **2005**, *353*, 109-116.

[7] a) X. Ren, D. J. Giesen, M. Rajeswaran, M. Madaras, *Organometallics* **2009**, *28*, 6079-6089; b) T. P. Brewster, J. D. Blakemore, N. D. Schley, C. D. Incarvito, N. Hazari, G. W. Brudvig, R. H. Crabtree, *Organometallics* **2011**, *30*, 965-973.

[8] J. M. Hearn, I. Romero-Canelón, B. Qamar, Z. Liu, I. Hands-Portman, P. J. Sadler, *ACS Chem. Biol.* **2013**, *8*, 1335-1343.
